# ‘AND’ logic gates at work: Crystal structure of Rad53 bound to Dbf4 and Cdc7

**DOI:** 10.1038/srep34237

**Published:** 2016-09-29

**Authors:** Ahmad W. Almawi, Lindsay A. Matthews, Larasati, Polina Myrox, Stephen Boulton, Christine Lai, Trevor Moraes, Giuseppe Melacini, Rodolfo Ghirlando, Bernard P. Duncker, Alba Guarné

**Affiliations:** 1 Department of Biochemistry and Biomedical Sciences, ON, Canada; 2 Department of Biology, University of Waterloo, Waterloo, ON, Canada; 3 Department of Chemistry and Chemical Biology, McMaster University, ON, Canada; 4 Department of Biochemistry, University of Toronto, Toronto, Canada; 5 Laboratory of Molecular Biology, National Institute of Diabetes and Digestive and Kidney Diseases, National Institutes of Health, Bethesda, MD, USA

## Abstract

Forkhead-associated (FHA) domains are phosphopeptide recognition modules found in many signaling proteins. The *Saccharomyces cerevisiae* protein kinase Rad53 is a key regulator of the DNA damage checkpoint and uses its two FHA domains to interact with multiple binding partners during the checkpoint response. One of these binding partners is the Dbf4-dependent kinase (DDK), a heterodimer composed of the Cdc7 kinase and its regulatory subunit Dbf4. Binding of Rad53 to DDK, through its N-terminal FHA (FHA1) domain, ultimately inhibits DDK kinase activity, thereby preventing firing of late origins. We have previously found that the FHA1 domain of Rad53 binds simultaneously to Dbf4 and a phosphoepitope, suggesting that this domain functions as an ‘AND’ logic gate. Here, we present the crystal structures of the FHA1 domain of Rad53 bound to Dbf4, in the presence and absence of a Cdc7 phosphorylated peptide. Our results reveal how the FHA1 uses a canonical binding interface to recognize the Cdc7 phosphopeptide and a non-canonical interface to bind Dbf4. Based on these data we propose a mechanism to explain how Rad53 enhances the specificity of FHA1-mediated transient interactions.

Stress generated during DNA replication is one of the biggest hurdles proliferating cells face to preserve genome integrity. Therefore, eukaryotic cells have conserved surveillance mechanisms, known as cell cycle checkpoints, to detect and repair damage generated during DNA replication^1^,^2^,^3^,^4^. Rad53, and its mammalian ortholog the checkpoint kinase 2 (Chk2), are key effector kinases of the DNA replication checkpoint^5^,^6^. Loss-of-function mutations in RAD53 cause loss of viability due to an essential function in maintaining dNTP levels during DNA replication, while hypomorphic RAD53 mutations result in DNA damage sensitivity and deficits in checkpoint responses^7^,^8^,^9^,^10^. Similarly, loss-of-function mutations in Chk2 lead to a defective checkpoint response^11^,^12^.

Rad53 contains two forkhead-associated (FHA) domains, as well as two SQ/TQ cluster domains (SCD), flanking its kinase domain. FHA domains are commonly found in DNA damage response proteins and mediate protein-protein interactions by recognizing phosphorylated epitopes on their binding partners^13^. During the replication checkpoint, phosphorylation-dependent interactions mediated by the FHA domains of Rad53 trigger hyperphosphorylation of the N-terminal SCD domain and lead to the full activation of Rad53^14^. It was generally believed that FHA domains recognize unstructured sequences containing a phosphorylated amino acid –often a threonine. Recent studies, however, have shown that FHA domains can also use alternate surfaces for protein oligomerization and to mediate protein-protein interactions^15^,^16^,^17^,^18^,^19^. The Dbf4-dependent kinase (DDK) and Rad9 are two binding partners of Rad53 during the replication checkpoint response. Dbf4 preferentially interacts with the N-terminal FHA domain (FHA1) of Rad53^20^, whereas Rad9 binds the C-terminal FHA domain (FHA2)^21^, reinforcing the idea that the two FHA domains recognize distinct features on their binding partners. DDK, a heterodimer composed of the Ser/Thr kinase Cdc7 and its regulatory subunit Dbf4, is one of the kinases known to hyperphosphorylate Rad53^22^. Reciprocally, Rad53 phosphorylates DDK to inhibit its activity, thereby preventing the firing of late replication origins^23^. This is crucial as it inhibits S-phase progression and allows cells to recover from replication stress.

The interaction between Rad53 and DDK is of special interest because it involves multiple interfaces of the FHA1 domain. The phosphoepitope-binding site recognizes an epitope present in DDK^24^, whereas one of the lateral surfaces of FHA1 interacts with the modified BRCT domain of Dbf4^16^, herein referred to as HBRCT domain due to the presence of an additional α-helix at the N-terminus of the domain. However, like many other relevant signaling interactions, the Rad53 and Dbf4 association is weak and presumably transient. The latter are especially difficult to study for effector proteins like Rad53, because they often interact with multiple partners using a common interface. To understand how Rad53 manages its multiple interactions during the steps leading to Cdc7 inhibition, we stabilized the Rad53:Dbf4 complex using glycine-rich linkers. We generated chimeras expressing the HBRCT (Dbf4) and FHA1 (Rad53) domains in tandem and solved the crystal structures of these chimeras in the absence and presence of a phosphorylated epitope derived from Cdc7. These are the first structures of an FHA domain bound to a binding partner through a non-canonical interface and they reveal a unique bipartite interface between Rad53 and Dbf4 that provides exquisite specificity despite the minimal interaction surface.

## Experimental Procedures

### Cloning and Expression

Dbf4-Rad53 chimeras were created by subcloning a codon-optimized fragment of Dbf4 encompassing amino acids 105–220^16^ followed by the FHA1 domain of Rad53 (amino acids 22–162) into a modified pET15b vector including His_6_-SUMO tag with a TEV protease cleavage site (pAG8586). The two protein fragments were connected directly (Dbf4(0)Rad53) or separated by a five-residue linker (Dbf4-SGASG-Rad53, herein referred to as Dbf4(5)Rad53). Clones were confirmed by DNA sequencing (MOBIX, McMaster University). Plasmids encoding the Dbf4(0)Rad53 (pAG8801) and Dbf4(5)Rad53 (pAG8805) chimeras were transformed in BL21(DE3) cells containing a plasmid encoding rare tRNAs. Cultures were grown to A_600_ = 0.7, induced by addition of 1 mM isopropyl β-D-1-thiogalactopyranoside, and incubated overnight at 16 °C with orbital agitation.

### Protein Purification

Cell pellets were resuspended in buffer A (20 mM TRIS-HCl pH 8.0, 500 mM NaCl, 1.4 mM 2-mercaptoethanol, 5% glycerol) and lysed by sonication. Lysates were cleared by centrifugation at 39,000 *g,* and the supernatants were loaded onto a HiTrap nickel-chelating HP column (GE Healthcare) equilibrated with buffer A. The His_6_-SUMO-tagged chimeras were eluted with a linear gradient to 300 mM imidazole. The fractions containing the chimera were pooled and injected onto a HiPrep 26/10 desalting column (GE Healthcare) equilibrated with buffer B (20 mM TRIS-HCl pH 8.0, 150 mM NaCl, 1.4 2-mercaptoethanol, 5% glycerol). The His_6_-SUMO tag was removed with tobacco etch virus (TEV) protease, and the tagless chimeras further purified by affinity (HiTrap nickel-chelating HP column, GE Healthcare) and size-exclusion chromatography (Superdex 75 (10/300) GL column, GE Healthcare). The purified proteins were concentrated to 9–12 mg/mL and stored in buffer B. Protein concentrations were determined using the Beer-Lambert equation with an extinction coefficient of 36,440 M^−1^ cm^−1^.

### Analytical ultracentrifugation

Sedimentation velocity experiments were conducted at 50,000 rpm and 20 °C on a Beckman Coulter ProteomeLab XLI analytical ultracentrifuge following standard protocols^25^. Samples of the Dbf4(5)Rad53 chimera were studied at various loading concentrations ranging from 2 to 310 μM in 0.2 M NaCl, 0.02 M TRIS-HCl pH 8.0, 1.4 mM 2-mercaptoethanol and 5% v/v glycerol. Samples were loaded in 2-channel centerpiece cells and data were collected using both the absorbance (280 nm) and Rayleigh interference (655 nm) optical detection systems when possible. Standard 12 mm centerpieces were used, whereas shorter 3 mm centerpieces were used for the higher concentration protein samples (>70 μM). Sedimentation data were time-corrected^26^ and analyzed in SEDFIT 15.01b^27^ in terms of a continuous c(*s*) distribution of sedimenting species with a resolution of 0.05 S and a maximum entropy regularization confidence level of 0.68. The solution density, solution viscosity and protein partial specific volume were calculated in SEDNTERP (ref. 28, http://sednterp.unh.edu/), and sedimentation coefficients *s* were corrected to standard conditions *s*_*20,w*_. Weighted-average sedimentation coefficients obtained by integration of the c(*s*) distributions were used to create an isotherm that was analyzed in SEDPHAT 13.0a in terms of a reversible monomer-dimer equilibrium. The protein extinction coefficient at 280 nm and the interference signal increment were calculated based on the amino acid composition in SEDFIT 15.01b^29^.

### Crystallization, structure determination and refinement

Crystals of the Dbf4(5)Rad53 grew in 50 mM sodium cacodylate pH 6.5, 12% PEG 4000 (v/v), and 250 mM MgCl_2_ and cryo-protected by addition of 10% ethylene glycol. A complete data set was collected at the X29 beamline of NSLS-I (Brookhaven National Laboratory). Data was processed and scaled in HKL2000 (see Table 1)^30^. A phosphorylated peptide (pPEP) derived from Cdc7 (^480^DGESpTDEDDVVS^491^) was purchased from GenScript and resuspended in buffer B. The Dbf4(0)Rad53 chimera was mixed with the phosphorylated peptide at a 10-fold molar excess and incubated at 4 °C for one hour prior to crystallization trials. Crystals of the Dbf4(0)Rad53-pPEP complex were grown in 100 mM TRIS pH 8.5, and 12.5% PEG 3350 (v/v) and cryo-protected by addition of 15% glycerol. A complete data set was collected at the O8B1-1 beamline of the Canadian Light Source and processed using XDS (see Table 1)^31^.

Both structures were determined by molecular replacement using the FHA1 domain of Rad53 (PDB 1G6G) and the HBRCT domain of Dbf4 (PDB 3QBZ) as search models. The initial models were refined by iterative cycles of manual model building in Coot and refinement in PHENIX^32^. The refined models have 98% (Dbf4(5)Rad53) and 98.4% (Dbf4(0)Rad53-pPEP) of the residues in the most favored regions of the Ramachandran plot and none in the disallowed regions. Quantitative analysis of the Dbf4(L)Rad53 (±pPEP) interfaces was done using the online Protein Interfaces Structures and Assemblies (PISA) server^33^. Figures showing molecular structures were generated using PyMOL^34^.

### Analysis of the NMR data

Gradient and sensitivity enhanced [^1^H-^15^N] heteronuclear single quantum coherence (HSQC) spectra were acquired at 306 K using a Bruker AV-700 MHz spectrometer equipped with a 5 mm TCI cryoprobe. Samples were prepared as described by ref. 16 with an equimolar concentration of FHA1 and HBRCT in either the absence or presence of 200 μM phosphorylated Cdc7 peptide (pPEP, ^480^DGES**pT**DEDDVVS^491^). Spectra were processed using NMRPipe^35^ and analyzed in Sparky.

### Yeast two-hybrid experiments

Point mutations within FHA1 (F146A and N112A/F146A) were generated by site-directed mutagenesis using pJG4-6 FHA1 (including residues 1–165 of Rad53) as the template. Single point mutations on Dbf4 (Y198A and K200A) were generated from the pEG202-Dbf4 full-length template. All constructs were verified by DNA sequencing (MOBIX, McMaster University). Two-hybrid analysis was carried out as described previously using pEG202-Dbf4-FL as the bait and the indicated pJG4-6 FHA1 variants as the prey^36^. The *lacZ* reporter pSH18-34, pEG-202-Dbf4-FL-derived bait and pJG4-6-Rad53-FHA1-derived prey plasmids were transformed into DY-1 yeast strain. Cells were grown to a concentration of 2 × 10^7^ cells/mL in Synthetic Defined (SD) medium lacking uracil, histidine, and tryptophan. Prey expression was induced for 5–6 h in 20 mL of 2% galactose-1% raffinose medium (BD Bioscience). Using a total of 5 × 10^6^ cells, the interactions between fusion proteins were detected by the quantitative β-Galactosidase (β-Gal) assay. The β-Gal activity was calculated using the following equation: β*-*Gal activity = 1,000 × *A*_420_/(*t* × *V* × *A*_600_), where *t* = time of reaction (in min) and *V* = volume of culture used in the assay (in mL). The remaining culture was centrifuged at 2,000 *g* for 3 min; the cell pellets were resuspended in lysis buffer (10 mM Tris-HCl pH 8.0, 140 mM NaCl, 1 mM EDTA, 1% Triton X-100, with protease inhibitors) and lysed with a bead beater (BioSpec Products, Inc.). Cell lysates were clarified by centrifugation and the extracted supernatant represented the whole-cell extract. Protein concentration was quantified using a Bradford assay, and protein expression was analyzed by Western blotting. The LexA-tagged Dbf4 bait was detected using a rabbit polyclonal anti-LexA antibody (Invitrogen), and the HA-tagged FHA1 prey was detected using a mouse monoclonal anti-HA antibody (Sigma). Alexa Fluor 647 goat anti-rabbit and Alexa Fluor 488 goat anti-mouse polyclonal secondary antibodies (Molecular Probes) were used.

## Results

### The Dbf4(L)Rad53 chimeras have a weak self-association

**Figure 1:**
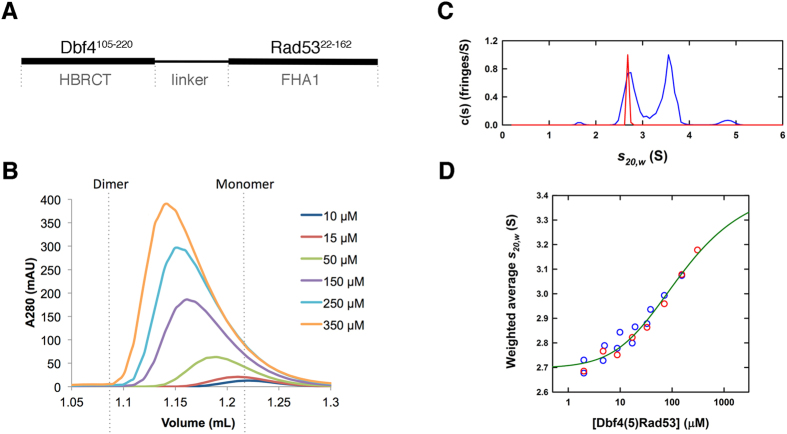
The Rad53(5)Dbf4 chimera exists in a monomer-dimer equilibrium. (**A**) Cartoon depicting how the Rad53(L)Dbf4 chimeras were generated. (**B**) Size exclusion chromatography profiles of Dbf4(5)Rad53 at increasing protein concentrations. Elution volumes for a ideal Dbf4(5)Rad53 monomer and dimer are indicated as dashed lines. (**C**) Normalized interference c(*s*) profiles for Dbf4(5)Rad53 at 2 μM (red) and 310 μM (blue) supporting a reversible monomer-dimer equilibrium. (**D**) Dependence of the weighted-average *s*_*20,w*_ on the loading concentration for absorbance (blue) and interference (red) sedimentation velocity data, along with the global best-fit monomer-dimer isotherm (green).

We have previously shown that the HBRCT domain of Dbf4, consisting of a BRCT fold immediately preceded by a helix, is necessary and sufficient for the interaction with the FHA1 domain of Rad53^36^. The interaction with this domain of Dbf4 is mediated by one of the lateral surfaces of the FHA1 domain rather than its phosphopeptide-binding pocket^16^. However, the instability of the HBRCT domain at high concentrations prevented the characterization of the reciprocal surface in Dbf4. Based on our biochemical, genetic and structural data, we generated a preliminary model of the Dbf4-Rad53 complex using the Rosetta software. In this model, the lateral surface of the FHA1 domain interacted with the concave surface of the HBRCT domain of Dbf4 leaving the termini of both domains in close proximity. Therefore, we anticipated that we could stabilize the interaction by producing the two domains as a single polypeptide chain. We fused the FHA1 domain of Rad53 at the C-terminus of the HBRCT domain of Dbf4 directly, or using a five-residue glycine/serine-rich linkers (Dbf4(0)Rad53 and Dbf4(5)Rad53; Fig. 1A). The resulting chimeras were monodisperse and behaved as monomers in solution as judged by dynamic light scattering and size exclusion chromatography. Despite being predominantly monomeric, the elution times from an analytical size exclusion chromatography varied in a concentration-dependent manner suggesting a weak intermolecular association (Fig. 1B and Supplementary Figure 1).

We carried out a series of sedimentation velocity experiments on the Dbf4(5)Rad53 chimera at increasing loading concentrations. The sedimentation experiments demonstrated the absence of very large species and yielded c(*s*) profiles that supported a reversible monomer-dimer equilibrium (Fig. 1C). Dimerization of chimeric proteins is not uncommon and it indicates that the two components of the chimera associate intermolecularly^37^,^38^,^39^. A weighted-average sedimentation coefficient isotherm was constructed and analyzed in terms of reversible monomer-dimer equilibrium (Fig. 1D), to obtain a dissociation constant K_d_ of 130 μM. As the isotherm does not adequately cover the high concentration region there may be significant errors in this value. Based on the reduced chi-squared, using the method of F-statistics^40^, we estimate 68% and 95% confidence limits of the K_d_ to be 70–260 μM and 50–400 μM. These values indicate the order of magnitude of the interaction and confirm that the HBRCT domain of Dbf4 and the FHA1 domain of Rad53 associate weakly.

### The Dbf4(L)Rad53 chimeras recreate the Dbf4:Rad53 interaction

To avoid constraints imposed by the presence of the linker joining the two proteins, we set crystallization trials of two chimeras: Dbf4(0)Rad53 and Dbf4(5)Rad53. The chimera containing a five-residue linker readily yielded diffraction-quality crystals (Table 1). The asymmetric unit contained four copies of the Dbf4(5)Rad53 chimera arranged to form four Dbf4:Rad53 complexes. The C-terminal end of Dbf4 (residues 216–220) and the N-terminal end of Rad53 (residues 22–29), as well as the five amino acid linker, were disordered in the structure (Supplementary Figure 2). This results in almost twenty amino acids missing in each polypeptide chain. The distance between the last ordered residue of the HBRCT domain of Dbf4 and the first ordered residue in the closest FHA1 neighbors, the crystal packing contacts and the behavior in solution of the chimeras, confirms that the Dbf4:Rad53 complex forms inter-molecularly. Importantly, the four complexes in the asymmetric unit had identical interfaces, indicating that the linkers did not constrain complex formation.

**Figure 2:**
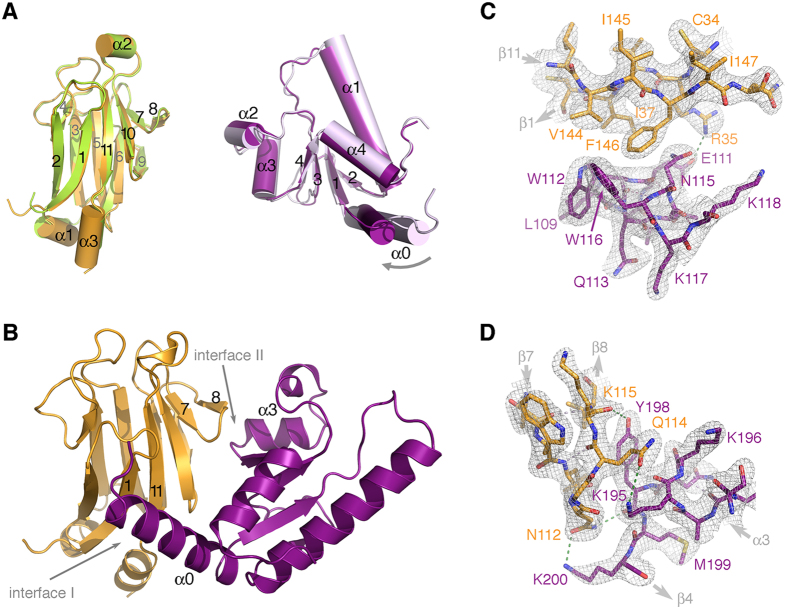
Structure of the Rad53(5)Dbf4 chimera. (**A**) Comparison of the structures of the FHA1 domain of Rad53 and the HBRCT domain of Dbf4 when crystallized on their own (PDB ID: 1G6G and 3QBZ) or forming a complex. The FHA1 domain is shown in green (1G6G) or gold (complex) and the HBRCT domain is shown in lilac (3QBZ) or purple (complex) with secondary structure elements labeled for clarity. (**B**) Ribbon diagram of the crystal structure of the Rad53:Dbf4 complex with Rad53 colored gold and Dbf4 colored purple. The interfaces mediating the complex, as well as the pThr-binding groove, are labeled. Detailed views of the interactions defining interface I (**C**) and interface II (**D**). Rad53 and Dbf4 residues are shown as sticks colored as in (**B**) and labeled. Refined 2Fo-Fc electron density maps are shown as a grey mesh contoured at σ = 1.2. Hydrogen bonds are shown as dashed lines.

The FHA1 and HBRCT domains have identical architectures in the complex as in their unbound structures (Fig. 2A). However, the helix α0 of the HBRCT domain swivels about twenty degrees upon complex formation (Fig. 2A). In good agreement with our previous results showing that the pThr-binding pocket of the FHA1 domain does not mediate the interaction with Dbf4^16^, the complex forms through the lateral surface of the FHA1 domain defined by the β2-β1-β11-β10-β7-β8 strands and the concave surface of the HBRCT domain (Fig. 2B). This interface, however, is quite limited because Dbf4 only contacts two small regions on each side of the lateral surface of Rad53. On one side of the interface, the side chains of residues Arg35 (β1), Ile37 (β1), Val144 (β11) and Phe146 (β11) of Rad53 are cradled by the α0 helix of the HBRCT domain, specifically by residues Glu111, Trp112, Asn115 and Trp116, defining interface I (Fig. 2C). On the other side, the loop containing residues Tyr198 and Lys200 of Dbf4 wraps around the β7/β8 loop of the FHA1 domain enabling the interaction between the amine group of Lys200 and Asn112 (β7) in Rad53 defining interface II (Fig. 2D). Globally the two interfaces bury a mere 10% of the total accessible surface area of the FHA1 (755 out of 6,664 Å^2^) and the HBRCT (801 out of 7,799 Å^2^) domains, a value that is below the cutoff for specific interactions as judged using the PISA server^33^. This is not surprising in light of the dissociation constant estimated from the sedimentation velocity and NMR analysis (Fig. 1 and ref. 16).

### Rad53 and Dbf4 contribute asymmetrically to the interface of the complex

The residues of the FHA1 domain mediating the interaction with Dbf4 in the crystal structure of the Dbf4(5)Rad53 chimera are the same as those previously identified using NMR^16^. Our previous work, however, could not explain why multiple point mutations on the FHA1 surface were required to abrogate complex formation^16^. These results were intriguing because Rad53 and Dbf4 interact with low affinity and, hence, we had not anticipated the need of multiple mutations to abrogate the interaction. Since the point mutations in Rad53 were designed based on sequence conservation, we had a better sampling of interface I than interface II (Fig. 2). Therefore, we decided to dissect the contributions of both interfaces to the complex formation.

**Figure 3:**
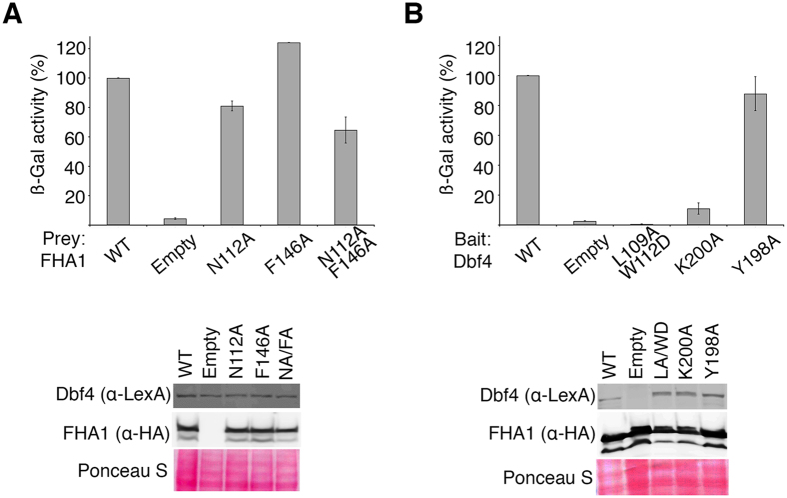
Two discrete interfaces contribute to the Rad53:Dbf4 interaction. (**A**) Yeast two-hybrid analysis using wild type Dbf4 as the bait and variants of the FHA1 domain of Rad53 as the preys. (**B**) Yeast two-hybrid analysis using variants of Dbf4 as the baits and the wild type FHA1 domain of Rad53 as the prey. In each case, the interaction is shown as a percentage of β-galactosidase activity for the interaction between wild-type proteins and represents the mean of three independent measurements (error bars represent S.D). Bait and prey expression levels were analyzed by Western blotting and relative protein loading assessed by Ponceau S staining. See Supplementary Figure 3 for original gels/blots.

We generated single point mutations in the FHA1 domain affecting either interface I (Phe146Ala) or interface II (Asn112Ala), as well as a double point mutation affecting both interfaces (Asn112Ala/Phe146Ala). We then measured the ability of these variants to interact with full-length Dbf4 using a yeast two-hybrid assay. As we expected from our previous work, the FHA1-Asn112Ala had a mild, yet significant, binding defect (Fig. 3A and Supplementary Figure 3A). Conversely, the FHA1-Phe146Ala variant interacted with Dbf4 better than wild type FHA1 suggesting that a smaller side chain at this position may help accommodate helix α0 of the HBRCT. The combination of both changes had a stronger defect than the FHA1-Asn112Ala variant, but retained about half of the residual binding to Dbf4 (Fig. 3A). When we conducted the reciprocal experiment, the results were more drastic. The Dbf4-Leu109Ala/Trp112Asp variant (affecting interface I) completely abrogated the interaction with the FHA1 domain, whereas variants affecting interface II had wide-ranging effects (Fig. 3B). On our structure, the loop containing residues Tyr198 and Lys200 of Dbf4 wraps around the β7/β8 loop of the FHA1 domain enabling the interaction with Asn112 (β7) in Rad53 (Fig. 2D). Mutation of Tyr198Ala did not affect the interaction with the FHA1 domain, whereas mutation of Lys200Ala disrupted binding to the FHA1 domain (Fig. 3B and Supplementary Figure 3B). Collectively these data suggest that hydrophobic contacts and the relative rigid body movement of helix α0 drive the interaction at interface I, whereas polar interactions determine interface II. Furthermore, they confirm that Dbf4 and Rad53 do not contribute equally to each interface, but both contact points are important for complex formation.

### Rad53 interacts simultaneously with Dbf4 and a phosphorylated peptide

The combination of hydrophobic and polar interactions segregated in two different contact areas could provide the means to regulate complex formation upon binding of additional partners. Since a phosphorylated binding epitope is necessary for the interaction of Rad53 with DDK, we sought to determine the structure of the Dbf4(L)Rad53 chimera bound to a phosphorylated peptide (Table 1).

**Figure 4:**
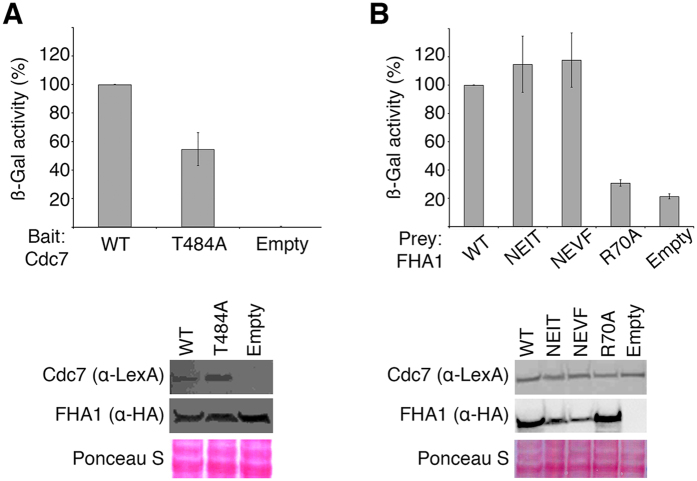
Rad53 recognizes a phosphorylated epitope in the Cdc7 subunit of the DDK complex. (**A**) Yeast two-hybrid analysis using either wild type or a T484A variant of Cdc7 as the baits and wild type FHA1 domain of Rad53 as the prey. (**B**) Yeast two-hybrid analysis using wild type Cdc7 as the bait and variants of the FHA1 domain of Rad53 as the prey. Bait and prey expression levels were analyzed by Western blotting and relative protein loading assessed by Ponceau S staining. See Supplementary Figure 4 for original gels/blots.

The fragment of Cdc7 encompassing residues 294–493 interacts reproducibly with the FHA1 domain of Rad53^24^. This region only contains one TXXD motif (^484^TDED^487^) that is conserved and has high phosphorylation probability^24^. In good agreement, a Cdc7 variant encompassing a Thr484Ala point mutation reduces the interaction of Cdc7 with the FHA1 domain of Rad53 to 50% of wild type (Fig. 4A and Supplementary Figure 4A). Reciprocally, a variant of Rad53-FHA1 unable to bind phosphorylated targets (FHA1-Arg70Ala) abrogates the interaction with Cdc7 (Fig. 4B and Supplementary Figure 4B). The differences between the Cdc7-Thr484Ala and Rad53-Arg70Ala variants suggest that Rad53 may be able to bind other epitopes in Cdc7 in the absence of Thr484.

**Figure 5:**
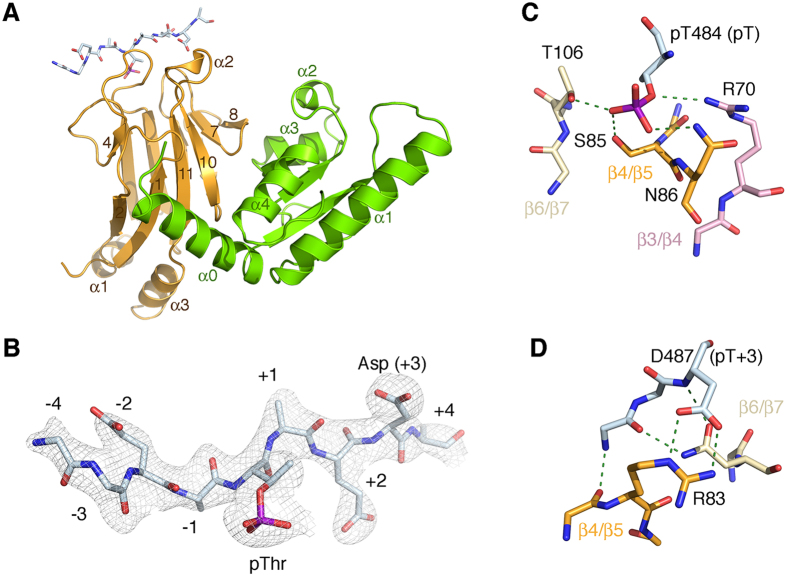
Structure of the Rad53:Dbf4:Cdc7 ternary complex. (**A**) Ribbon representation of the ternary complex on a similar view as in Fig. 2. Rad53 (orange) and Dbf4 (green) are shown as ribbons. The Cdc7-derived peptide (pPEP) is shown as colored coded sticks. (**B**) Detail of the electron density map around the phosphorylated peptide shown as a grey mesh contoured at σ = 1.0. (**C**) Detail of the hydrogen-bond network stabilizing pThr484. (**D**) Detail of the hydrogen bond interactions defining the specificity at the pT + 3 position of the peptide, as well as additional hydrogen bonds stabilizing the main chain of the peptide. Hydrogen bonds are shown as green dashed lines.

Conversely, variants disrupting the Rad53:Dbf4 interface do not affect binding to Cdc7 (Fig. 4B and ref. 16). Since we have previously shown that a peptide derived from this motif of Cdc7 (pPEP, ^480^DGES**pT**DEDDVVS^491^) binds to the FHA1 domain of Rad53 in a phosphorylation-dependent manner *in vitro*^16^, we used this peptide for subsequent crystallographic studies. Crystals of the ternary Rad53-Dbf4-Cdc7 complex grew in the P2_1_ space group and diffracted X-rays beyond 2.3 Å resolution. We determined the structure by molecular replacement using the structures of the individual FHA1 and HBRCT domains as search models. The molecular replacement solution showed well-defined electron density for the two domains, as well as the main chain and most side chains of the phosphorylated peptide (Fig. 5A,B). Similar to other structures of FHA1 domains bound to phosphorylated peptides, pPEP is bound at one end of the FHA1 domain and interacts with residues in the β3/β4, β4/β5 and β6/β7 loops^13^. The phosphate moiety of pThr484 is held in place through hydrogen bonds with Arg70, Ser85, Asn86 and Thr106 (Fig. 5C); the pT + 3 aspartate residue (Asp487) is anchored through a salt bridge with Arg83; and the main chain of the intervening residues is further stabilized through hydrogen-bonds with the main chain carbonyl of Ser82 and the side chain of Asn107 (Fig. 5D).

The Dbf4:Rad53 interface is similar, but not identical, to the binary complex. Superimposition of the FHA1 domains in the binary and ternary complexes revealed that the β1 strand and the β1/β2 loop were virtually invariant (r.m.s.d. <0.1 Å^2^). Therefore, we used this region of the FHA1 to superimpose and compare the two complexes. As expected, the loops defining the pThr-binding groove of the FHA1 domain had larger deviations (0.34 < r.m.s.d. <0.63), caused by the binding of the phosphopeptide. Conversely, the residues in FHA1 mediating the interaction with Dbf4 were barely affected by phosphopeptide binding (0.15 < r.m.s.d. <0.35).

**Figure 6:**
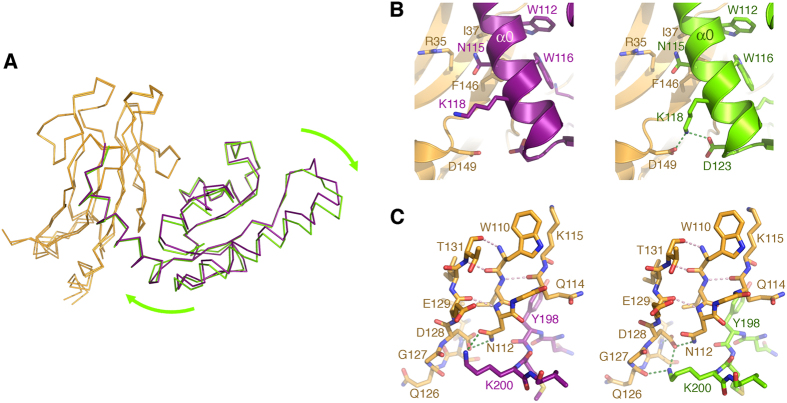
Peptide-binding induces a rigid body movement of Dbf4. (**A**) Opposite views of the Rad53:Dbf4:Cdc7 complex (gold-green) superimposed onto the Rad53:Dbf4 complex (gold-purple). (**B**) Detail of the different interactions of Lys118 (Dbf4) in in the binary (left) and ternary (right) complexes. (**C**) Detail of the conformational change imposed onto the side chain of Lys200 (Dbf4) upon binding of the phosphorylated peptide. In the binary complex (left-side panel) Lys200 interacts with Asn112 (Rad53), whereas in the ternary complex (right-side panel) interacts with Gly127 and Asp128 (Rad53).

Binding of the phosphopeptide, however, induces a small rigid body movement of Dbf4 around the two interfaces holding the complex (Fig. 6A). The HBRCT domain seesaws pushing helix α1 away from the FHA1 domain while pulling the α0/β1 loop towards the FHA1 domain. This rotation is identical for both complexes in the asymmetric unit and, though subtle, the movement is enough to reorganize some of the residues at both interfaces. Upon binding to pPEP, the side-chain of Lys118 (interface I in Dbf4) comes close to the side-chains of Asp123 (Dbf4) and Asp149 (Rad53) stabilizing the interaction of the C-terminus of helix α0 in Dbf4 with Rad53 (Fig. 6B). On the ternary complex, Lys200 (interface II in Dbf4) is not hydrogen-bonded to Asn112. Binding to pPEP pushes the α3/β4 loop of Dbf4 closer to the β10 strand where the new conformation of Lys200 is stabilized through hydrogen bonds with Gln126 and Asp128 (Fig. 6C).

### Phosphopeptide binding modulates the Rad53:Dbf4 interaction

Given the subtle movement of the HBRCT domain, the analysis of the two interfaces did not show significant differences in the extension of the interface or solvation energy (Table 2). Dbf4 had a minimal gain in solvation energy suggesting the HBRCT domain has more surface exposed residues in the ternary than the binary complexes (Table 2). The differences between the Dbf4:Rad53 interface in the binary and ternary complexes could indicate that binding of the phosphopeptide allosterically regulates the interaction. However, these differences could also be due to crystal packing environment or the different linker length of the fusions.

The asymmetric unit of the binary complex included four Dbf4(5)Rad53 molecules defining four Dbf4:Rad53 interfaces, whereas that of the ternary complex included two Dbf4(0)Rad53 molecules defining two Dbf4:Rad53 interfaces. Superimposition of each ternary complex onto any of the binary complexes revealed that the peptide moiety could only be accommodated in half of the complexes, explaining why crystals of the ternary complex grew in different conditions. However, no other crystal contacts mediated by the FHA1 or HBRCT domains enhanced or prevented the movement in Dbf4. Therefore, phosphopeptide binding rather than crystal packing is the likely driving force of the movement.

We have previously shown that losses of cross-peak intensity in the HSQC spectrum of ^15^N-labeled Rad53 upon binding Dbf4 serve as sensitive reporters to map the interface of the complex^16^. If phosphopeptide binding to the FHA1 domain weakened the interaction, we would expect an enhancement of cross-peak intensities for the Rad53 residues at the interface with Dbf4. Despite maintaining similar conformations in the binary and ternary crystal structures, two residues on interface I (Ile37 and Phe146) showed increased cross-peak intensities in the ternary complex (Supplementary Figure 5). These observations are consistent with the idea that phosphopeptide binding to the FHA1 domain weakens the Rad53:Dbf4 interaction. However, some surface residues beyond the complex interface also show variations of cross-peak intensities between the two complexes (Supplementary Figure 5). Interestingly, a number of residues within the hydrophobic core of Rad53 display decreased cross-peak intensities upon phosphopeptide binding (Supplementary Figure 5). These residues form a continuous network from the β3/β4 loop (Phe68-Gly69) to the β9/β10 loop (Leu124-Ser125) that propagates across the β-sheet defined by strands β4-β3-β5-β6-β9. Such intensity losses typically reflect changes in internal dynamics and could possibly reveal an allosteric network to report the presence of the phosphorylated peptide to the Rad53:Dbf4 interface. While these changes could explain how the two inputs of the logic gate sense each other to elicit a single output, the idea awaits further validation.

## Discussion

Yeast genetics has delineated the factors and hierarchy of interactions involved in the DNA damage response, but the molecular detail has remained elusive because most of the interactions driving the checkpoint response are transient. This problem is aggravated for ‘AND’ logic gates because they recognize two or more inputs to produce a single signal^41^,^42^,^43^, but disruption of any of the inputs disrupts the output leading to technically biased interpretations. We have found that the FHA1 domain of Rad53 functions as a ‘AND’ logic gate for its interaction with DDK, thereby explaining more than a decade of partly conflicting results^16^,^20^,^36^,^44^,^45^. The crystal structures of the FHA1 domain of Rad53 bound to one (HBRCT) or both (HBRCT and phosphoepitope) partners in the DDK complex presented here unveil how this logic gate simultaneously recognizes two inputs and provide the first image of an FHA domain recognizing a binding partner through a non-canonical interface.

The interaction of Rad53 with the DDK complex is reminiscent of the interaction between Chk2 (the human ortholog of Rad53) and BRCA1, where the tandem BRCT repeat of BRCA1 simultaneously recognizes two distal surfaces in the FHA domain of Chk2^46^. In the BRCA1:Chk2 complex, the interaction involves the pThr-binding site and a conserved hydrophobic patch on one of the lateral surfaces of the FHA domain. Disruption of either contact point prevents the interaction, and mutation of the hydrophobic patch has been linked to Li-Fraumeni syndrome^46^. However, Dbf4 and BRCA1 do not interact with the same lateral surface of the FHA domains of Rad53 and Chk2, exposing the extreme plasticity of FHA domains to enhance binding specificity.

Both Rad53 and Chk2 dimerize in solution and this is important to promote kinase activation by trans-autophosphorylation^47^,^48^. Dimerization is triggered by damage-induced phosphorylation of a threonine within the SCD of the kinase. The dimers associate in a face-to-face configuration that promotes the swap of the activation loops for phosphorylation in trans^47^,^49^. In the crystal structure of Chk2, one of the lateral surfaces of the FHA domain also contributes to the dimerization interface and, in fact, the requirement of a phosphorylated threonine residue is bypassed by protein overexpression indicating that the kinase and FHA mediated interactions suffice to stabilize the dimer^47^. The surface of the FHA domain involved in the dimerization interface is the same as that of Rad53 mediating the interaction with Dbf4 (Supplementary Figure 6). However, the two kinases transition to monomers to phosphorylate downstream targets^47^,^50^,^51^, implying that dimer formation and subsequent dissociation may determine the hierarchy of checkpoint events.

In contrast to the Chk2 dimer, where the entire lateral face of the FHA domain contributes to dimer formation, Dbf4 only contacts two points on the lateral face of the FHA1 domain. Interestingly, mutations on the two surfaces are not reciprocal indicating that each partner contributes asymmetrically to the two small interfaces mediating the interaction with Dbf4, thereby suggesting a sophisticated way to gain binding-specificity without strengthening the interaction. To our knowledge, this is the first crystal structure of an FHA domain bound to a binding partner through a non-canonical interface and lays the foundation to study how FHA domains can exploit canonical and non-canonical interactions to increase binding specificity of low-affinity interactions and, in turn, extend the functional repertoire of this phosphoepitope binding module.

## Additional Information

**Accession codes:** Coordinates and Structure factors for the binary and ternary complexes have been deposited as Protein Data Bank entries 5T2F and 5T2S.

**How to cite this article**: Almawi, A. W. *et al*. ‘AND’ logic gates at work: Crystal structure of Rad53 bound to Dbf4 and Cdc7. *Sci. Rep.* **6**, 34237; doi: 10.1038/srep34237 (2016).

## Supplementary Material

Supplementary Information

## Figures and Tables

**Table 1:** Data collection and refinement statistics.

	Dbf4(5)Rad53	Dbf4(0)Rad53 + pPEP
Data Collection		
Beamline	X29 (NSLS)	08B1-1 (CLS)
Wavelength (Å)	1.1	0.979
Space group	P 1	P 2_1_
Cell dimensions		
a, b, c	57.7, 66.6, 86.6	64.5, 87.3, 66.1
α, β, γ	109.5, 90.1, 90.1	90, 94, 90
Resolution	35–2.3 (2.34–2.3)	44.6–2.25 (2.31–2.25)
Reflections (total/unique)	887,843/55,038	101,589/36,580
Completeness (%)	87.2 (57.3)	98.3 (97.4)
CC1/2 (%)	97.1 (92.5)	99.3 (31.8)
I/σ (I)	13.6 (1.4)	8.15 (1)
Redundancy	1.6 (1.4)	2.8 (2.8)
Refinement		
Resolution (Å)	35–2.66	44.6–2.4
Completeness (%)	91.1	98.3
R_work_/R_free_ (%)	20.6/23.7	20.7/22.9
Atoms refined	15,338	8,312
Solvent Atoms	175	192
rmsd in bonds (Å)	0.004	0.003
rmsd in angles (°)	0.834	0.733
Mean B values (Å^2^)	45.6	51.5

**Table 2:** PISA analysis of the Dbf4:Rad53 and Dbf4:Rad53:Cdc7 complexes.

Complex	Interface (Å^2^)	ΔG solvation (kcal/mol)	Solvation Energy contribution			
			Dbf4		Rad53	
			Structure	Average gain	Structure	Average gain
Rad53:Dbf4	684–707	−3.5 – −5.0	−96.3	−3.75	−115	−1.5
Rad53:Dbf4:Cdc7	684	−3.8	−96.7	−2.2	−115.1	−1.3
