# Supplementary material for: ‘AND’ logic gates at work: Crystal structure of Rad53 bound to Dbf4 and Cdc7
Source: Sci Rep. 2016 Sep 29;6:34237. doi: 10.1038/srep34237 (PMC5041073; doi:10.1038/srep34237)
Supplement: Supplementary Information [file srep34237-s1.pdf]

**Supplementary Material:**

# 'AND' logic gates at work: Crystal structure of Rad53 bound to Dbf4 and Cdc7

Ahmad W. Almawi[1], Lindsay A. Matthews[1,5], Larasati[3], Polina Myrox[3], Stephen Boulton[2], Christine Lai[4], Trevor Moraes[4], Giuseppe Melacini[2], Rodolfo Ghirlando[5], Bernard P. Duncker[3], Alba Guarné[1]

---

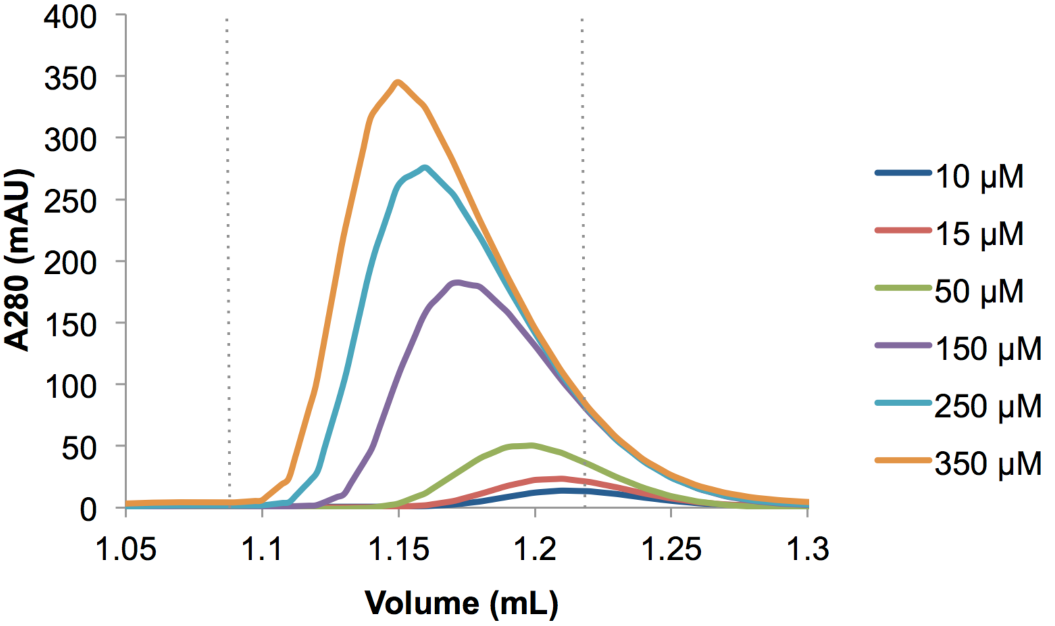


**Supplementary Figure 1: The Dbf4(0)Rad53 chimera exist in a monomer:dimer equilibrium.**

Size exclusion chromatography profiles of Dbf4(L0)Rad53 at increasing concentrations.

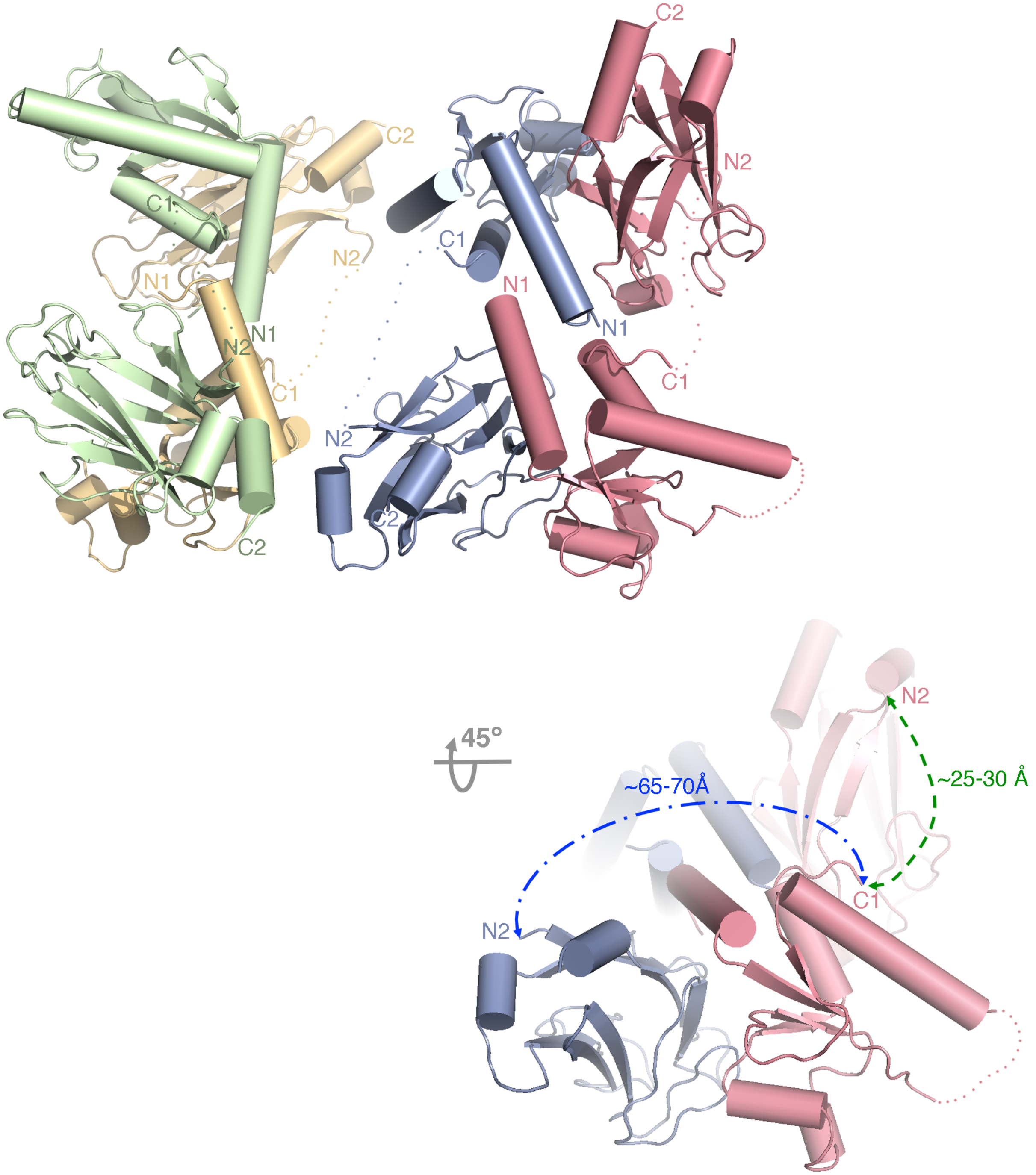


**Supplementary Figure 2: Crystal packing of the binary complex.** The four molecules in the asymmetric unit are shown color-coded with the N- and C-termini of the HBRCT and FHA1 domains labeled. The bottom panel shows the approximate distances between the C-terminus of the HBRCT and the N-terminus of its closest FHA1 neighbors.

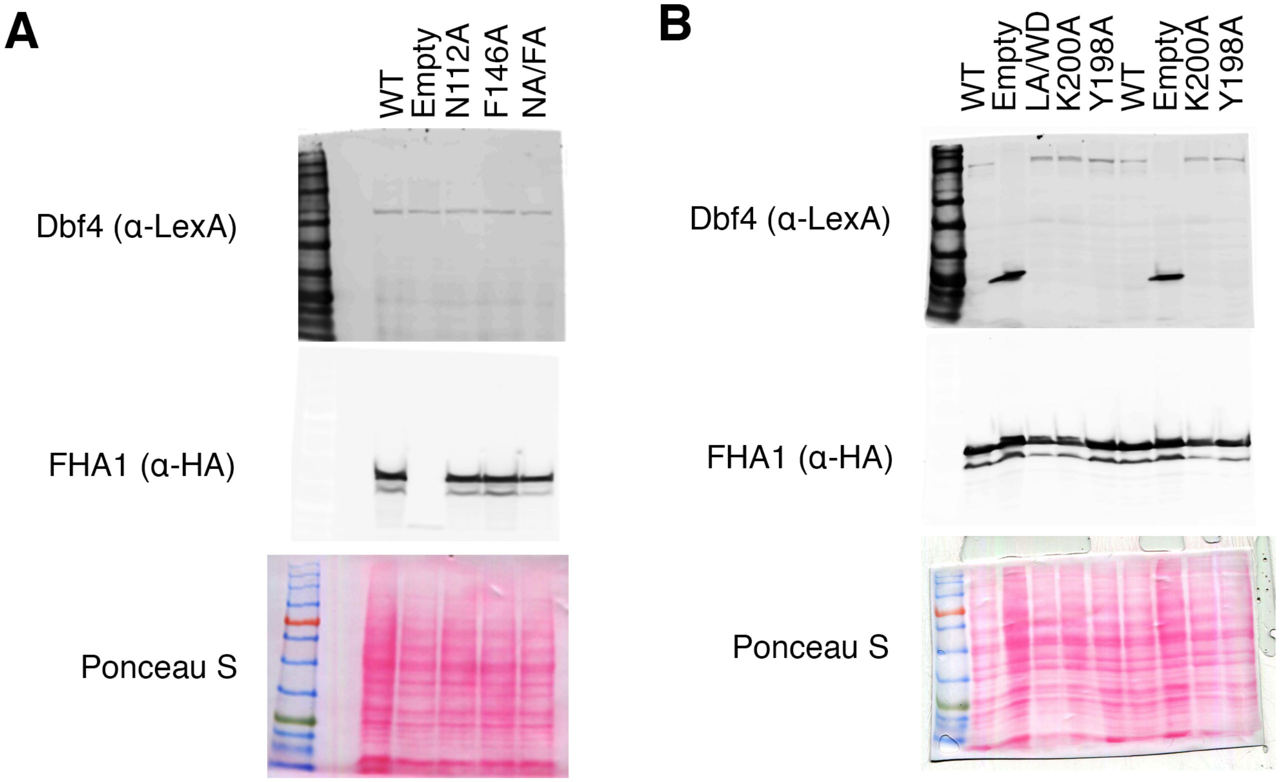


**Supplementary Figure 3: Original gels and blots for Figure 3.** To control for the two-hybrid bait and prey expression levels in **Figure 3**, whole cell extracts were prepared from transformants following prey induction and analyzed by Western blotting using rabbit anti-LexA antibody (bait) and mouse anti-HA monoclonal antibody (prey), along with Alexa Fluor 647-conjugated goat anti-rabbit and Alexa Fluor 488-conjugated goat anti-mouse secondary antibodies, respectively. Prior to detection, the membrane was stained with Ponceau S to assess relative protein loading. **(B)** Yeast two-hybrid analysis using variants of Dbf4 as the baits tested using the wild type FHA1 domain of Rad53 (left lanes) or the FHA1-F146A variant of Rad53 (right lanes) as the prey.

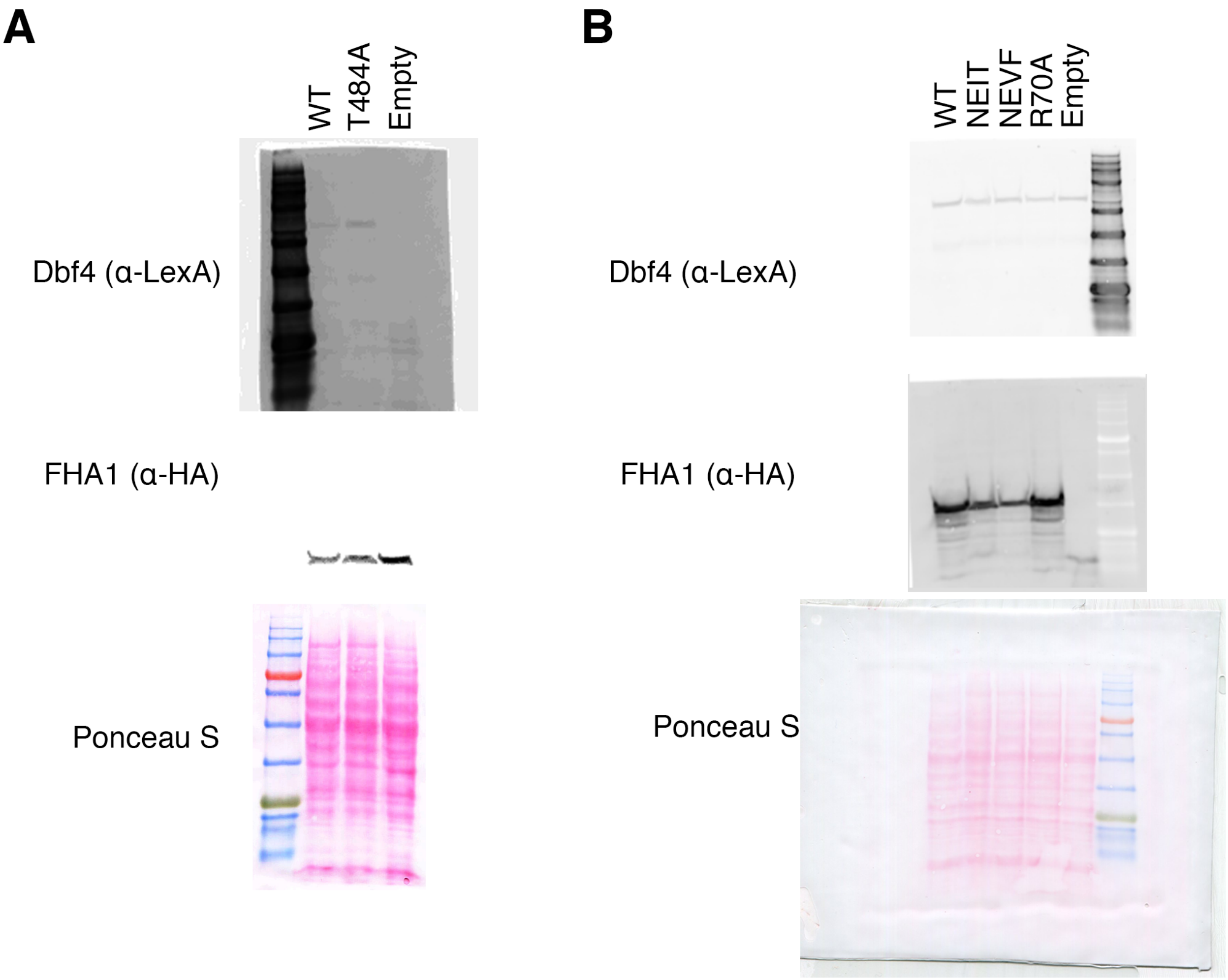


**Supplementary Figure 4: Original gels and blots for Figure 4.** To control for the two-hybrid bait and prey expression levels in **Figure 4**, whole cell extracts were prepared from transformants following prey induction and analyzed by Western blotting using rabbit anti-LexA antibody (bait) and mouse anti-HA monoclonal antibody (prey), along with Alexa Fluor 647-conjugated goat anti-rabbit and Alexa Fluor 488-conjugated goat anti-mouse secondary antibodies, respectively. Prior to detection, the membrane was stained with Ponceau S to assess relative protein loading.

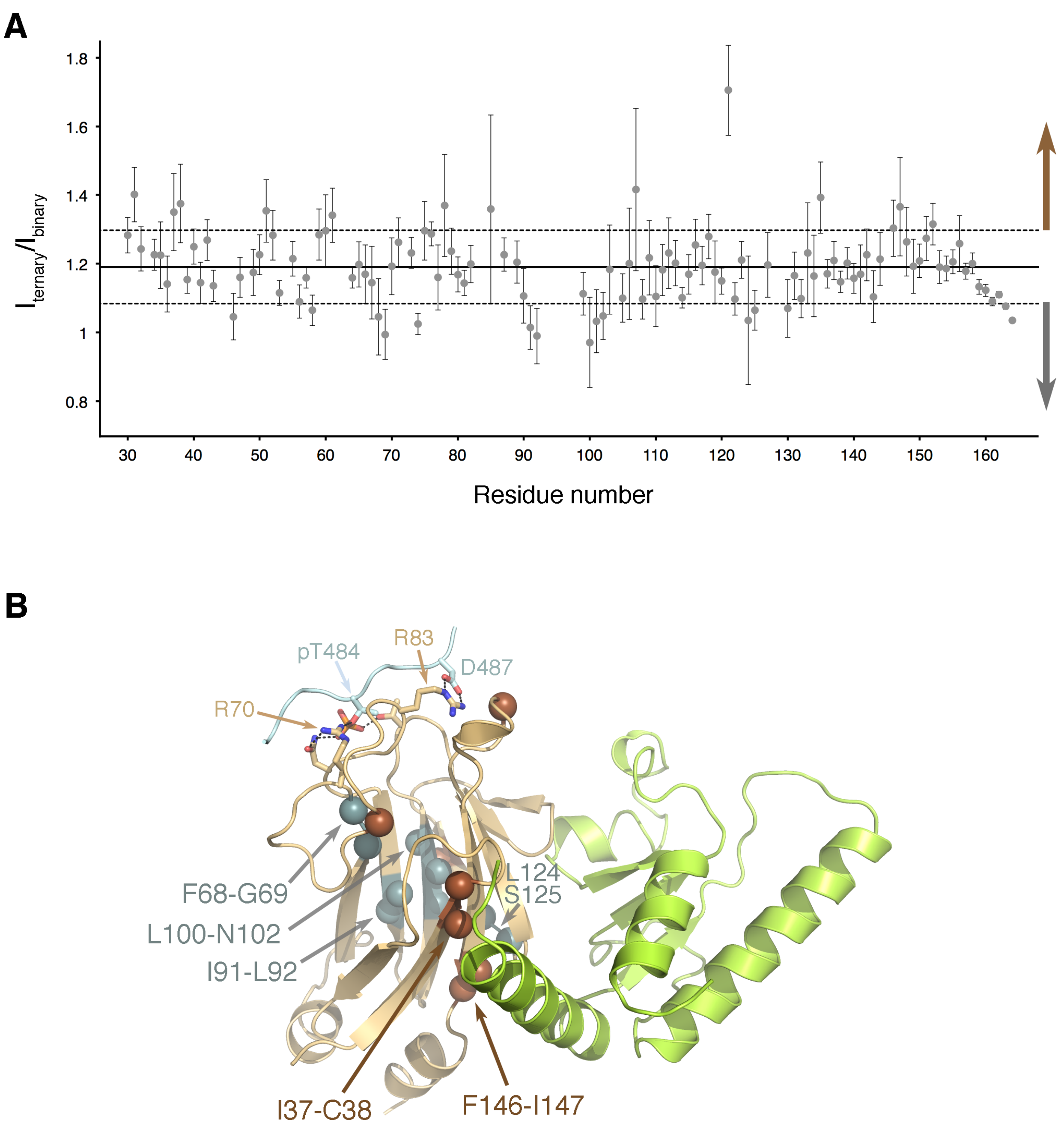


**Supplementary Figure 5: $^{15}$N-HSQC intensity changes confirm weakening of the Rad53:Dbf4 complex caused by binding of the phosphopeptide to Rad53. (A)** Plot of residue specific ratios between cross-peak intensities in the $^{15}$N-HSQC spectra of the binary (Rad53:Dbf4) and ternary (Rad53:Dbf4:Cdc7) complexes. The solid and dashed horizontal lines represent the average intensity ratio +/- one standard deviation. **(A)** Residues with values either greater or lower than the average +/- one standard deviation were plotted onto the X-ray structure of the ternary complex as brown or blue spheres, respectively **(B)**.

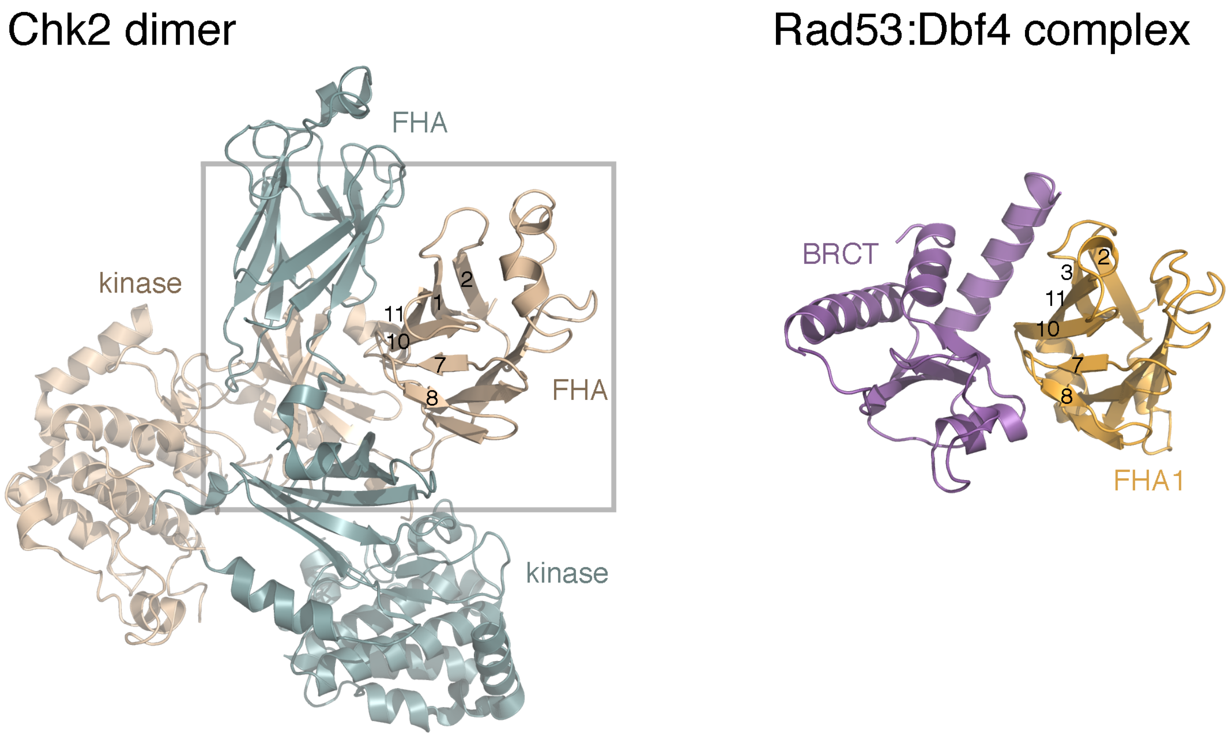


**Supplementary Figure 6: Interaction surfaces mediating dimerization of Chk2 and the Rad53:Dbf4 complex.** Ribbon diagram of the Chk2 crystal structure (left) showing with the two protomers of the dimer shown in steel blue and tan. The lateral surface of the FHA domain interacts with the FHA domain and the N-terminal lobe of the kinase domain on the second protomer. Comparison with the crystal structure of the Rad53:Dbf4 complex shows that the FHA1 domain of Rad53 (light orange) uses the equivalent lateral surface to interact with the BRCT domain of Dbf4 (purple).
